# Biomechanical effects of stitches on the intra-articular mid-substance of quadruple hamstring-tendon grafts for anterior cruciate ligament reconstruction – a pilot comparative cadaveric study

**DOI:** 10.1186/s43019-020-00059-y

**Published:** 2020-07-29

**Authors:** Maurise Saur, Philippe Clavert, François Bonnomet, Henri Favreau, Matthieu Ehlinger

**Affiliations:** 1 Service de Chirurgie Orthopédique et de Traumatologie, Hôpital de Hautepierre, Hôpitaux Universitaires de Strasbourg, 1 Avenue Molière, 67098 Strasbourg cedex, France; 2 Laboratoire iCube-GEBOAS, CNRS UMR 7357, Equipe 12 Matériaux Multi-échelles et Biomécanique, Institut de Mécanique des Fluides et des Solides, 2-4 Rue Boussingault, 67000 Strasbourg, France; 3 Institut d’Anatomie Normale et Pathologique de Strasbourg, 1 Place de L’Hôpital, 67000 Strasbourg, France

**Keywords:** Anterior cruciate ligament, Biomechanical testing, Quadruple hamstring-tendon graft, Graft preparation, ACL reconstruction, Load to failure test, Cadaveric study

## Abstract

**Background:**

There is little data in the literature regarding the preparation methods of the intra-articular portion of quadruple hamstring-tendon grafts for anterior cruciate ligament (ACL) reconstruction. The aim of this study was to compare the biomechanical properties of a sutured transplant to that of a non-sutured transplant. The hypothesis was that adding stitches to the intra-articular portion of the graft increased its resistance.

**Method:**

A comparative cadaveric study was carried out on five pairs of knees. The average age of the cadavers was 68 years. The exclusion criterion was past knee surgery. In the Sutured Group (SG) two stitches were made on the grafts. No stitches were made on the grafts of the Non-sutured Group (NSG). A tensile failure test was carried out using an Instron® loading machine. The maximal load to failure and stiffness were recorded and we observed the mode of failure for each graft. Statistical analysis was performed using the Wilcoxon rank sum test. Level of significance was set at *p* < 0.05.

**Results:**

The hypothesis proposed was not confirmed; adding stitches to the intra-articular portion of the four-strand hamstring-tendon graft does not increase its biomechanical properties. The maximal load to failure was 233.5 N ± 40.6 (186.7–274.5 N) for the NSG, 19.6% higher than for the SG which was 195.2 N ± 42.9 (139.0–238.2 N). Nevertheless, the difference observed was not statistically significant (*p* = 0.188). The stiffness of the grafts for the NSG was 23.5 N/mm ± 5.3 (17.8–29 N/mm) and 19.7 N/mm ± 5.5 (13.2–24.7 N/mm) for the SG grafts. Overall stiffness values for the NSG were 19% higher than those of the SG; however, the results were not statistically significant (*p* = 0.438). The failure mode was a rupture at the fixation point except for one sample from the SG which failed at an intra-articular stitch.

**Conclusion:**

Whilst the initial hypothesis was not verified, nevertheless, the maximal loads to failure and stiffness were approximately 20% higher when there were no intra-articular stitches compared to the situation where stitches were added to the intra-articular portion of the graft. This was a cadaveric pilot study and, therefore, whilst we are not able to extend our results to clinical practice, the outcomes would indicate the need for further development of this and related protocols deriving from the question of whether there is weakening the graft when adding stitches to its mid-substance. These results remain to be confirmed by further research.

## Introduction

Anterior cruciate ligament (ACL) reconstructions performed increase year on year and closer attention has been paid to revision cases in order to understand the reasons for re-rupture [1]. Several ACL reconstruction techniques are commonly currently used [2]. Whatever technique is applied, such transplants must meet a set of specifications in terms of graft strength and stiffness [3]. Current scientific data shows few biomechanical differences between the various types of transplants [4, 5]. However, other studies have brought to light that a variation of in-tunnel placement, or type of fixation could influence post-operative knee kinematics and loads [6, 7]. This suggests that there are multiple biomechanical factors that can influence rupture patterns.

Despite the existence of extensive literature on ACL reconstruction using hamstring-tendon grafts [8], there is no consensus on the best preparation methods of the intra-articular portion of the transplant.

Some surgeons do not add any stitches, whilst others join the four strands together with several stitches, or perform homogeneous suturing along the entire length of the transplant. There is no consensus and few publications regarding the preparation of the intra-articular portion of the four-strand hamstring transplant. To our knowledge, three articles have been published [6, 7] to date and none of them have analysed isolated additional stitches.

The aim of this study was to evaluate whether complementary stitches passing through the four-strands of the ‘central’ portion of the transplant, would increase its tolerable load to failure and stiffness. A cadaveric study was carried out to compare the biomechanical characteristics of a ‘sutured’ transplant to that of a ‘non-sutured’ transplant. The assumed cadaveric study hypothesis was that complementary stitches would increase the resistance of the hamstring autograft. The main outcome was the maximum load at failure of the graft and the secondary outcomes were stiffness and the failure mode.

## Method

### Study design

This cadaveric study was performed at the GEBOAS-ICube Biomechanics Laboratory of the Institute of Anatomy of Strasbourg, France. Five pairs of fresh-frozen cadaver legs (10 knees) were used. The donors were three men and two women with a mean age of 68 years (61–79 years). Knees with evidence of prior surgery were excluded from the study. None of the knees had visible incisions, ligament damage nor osteoarthritis [7].

The specimens were stored at − 20^°^ to preserve the mechanical properties of the tissues [4, 5]. Prior to preparation, the specimens were thawed for 12 h at room temperature (18 °C) [8]. The cadaver legs were cut at the junction of the proximal and middle thirds of the thigh and at the junction of the middle and distal thirds of the lower leg to harvest the entire semi-tendinosus and gracilis tendons.

**Fig. 1:**
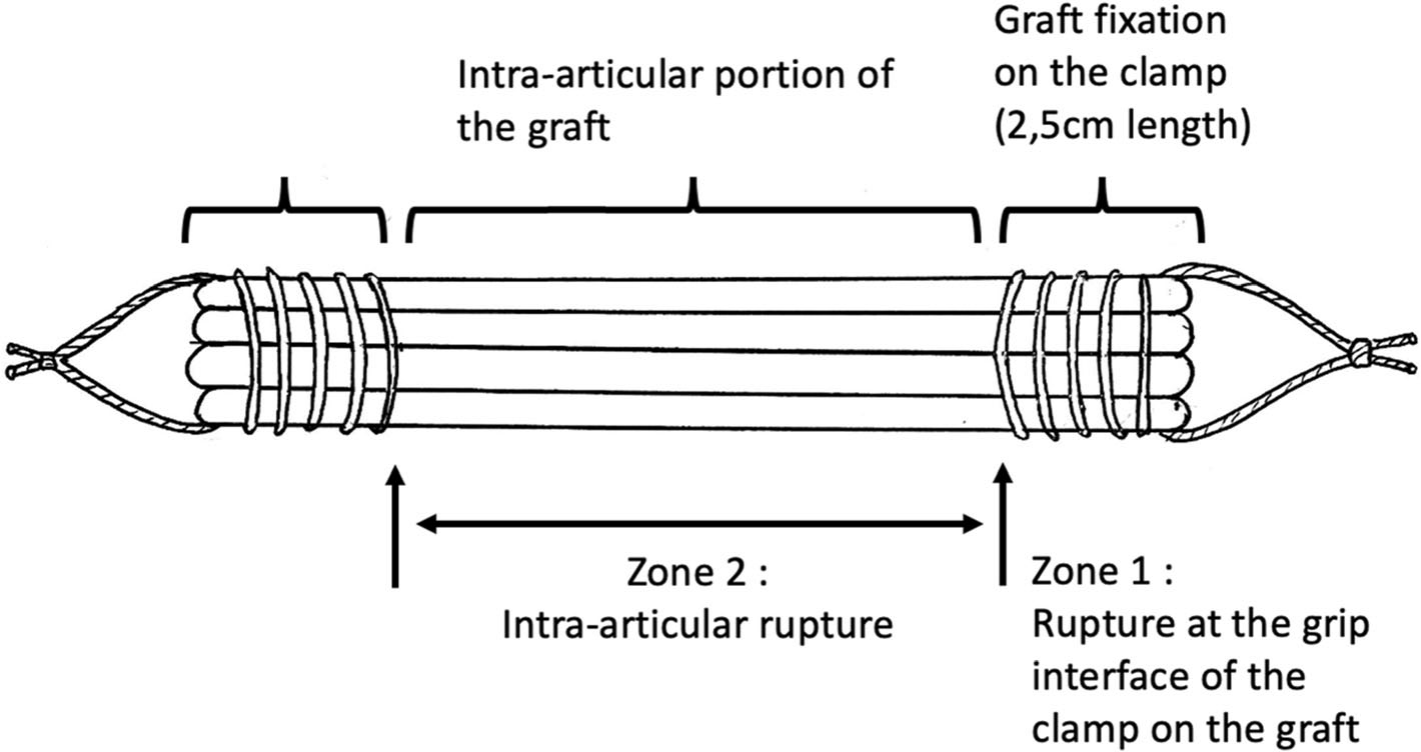
Four-strand hamstring-tendon graft

The preparation of the grafts was identical for every subject according to the surgical reference technique [2]. The semi-tendinosus and gracilis tendons were harvested with a stripper after extensive dissection. They were left attached to their tibial insertion. They were folded over to create a firm, four-strand transplant, then shaped into a tube by placing sutures over a length of 2.5 cm at each end (Fig. 1). Höher et al. [9] demonstrated that suturing the graft length that is used for fixation improves significantly its fixation strength. The tibial insertion pedicle was then cut to free the transplant for biomechanical testing. The mean length of the grafts was 70.3 mm [45–90] and the mean diameter was 9.2 mm [8–10]. There were no significant differences between the groups for both length and thickness.

**Fig. 2:**
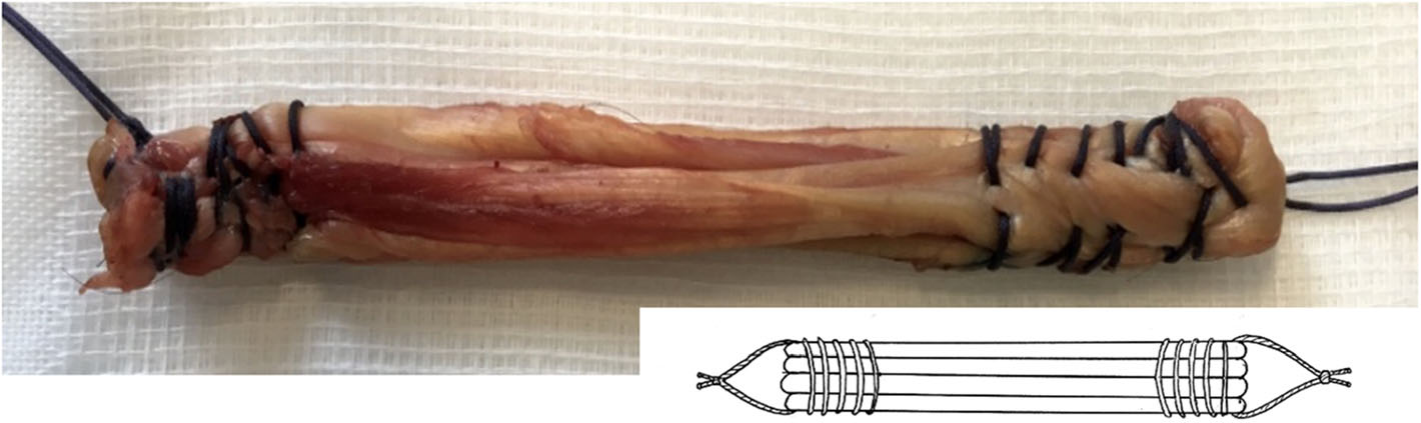
Non-Sutured Group transplant (NSG)

**Fig. 3:**
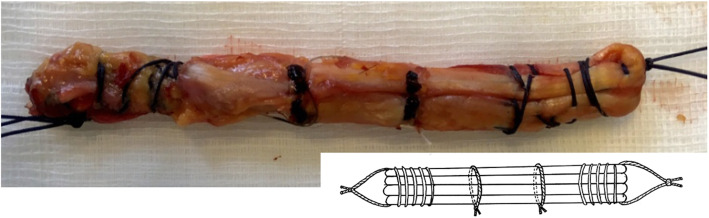
Sutured Group transplant (SG).

The knees were paired in order to create two groups. The first group was composed of all the right-sided knees on which no stitches were performed on the intra-articular portion: the Non-Sutured Group (NSG) (Fig. 2). The second group was the Sutured Group (SG) that comprised all the left-sided knees. Two stitches were made using a 3/0 braided and absorbable multi-filamentous thread (Vicryl^TM^, Ethicon®, Somerville, NJ, USA). The area between the two attachments of the graft ends was measured and divided into three parts. At the junction of each third, an additional stitch was applied which passed through each of the four strands (Fig. 3).

To prevent desiccation, the tendons were moistened and wrapped in saline-soaked gauze sponges. The sponges were then wrapped in aluminium foil, placed in an airtight bag, and stored at − 20 °C until further use [10].

### Mechanical testing

**Fig. 4:**
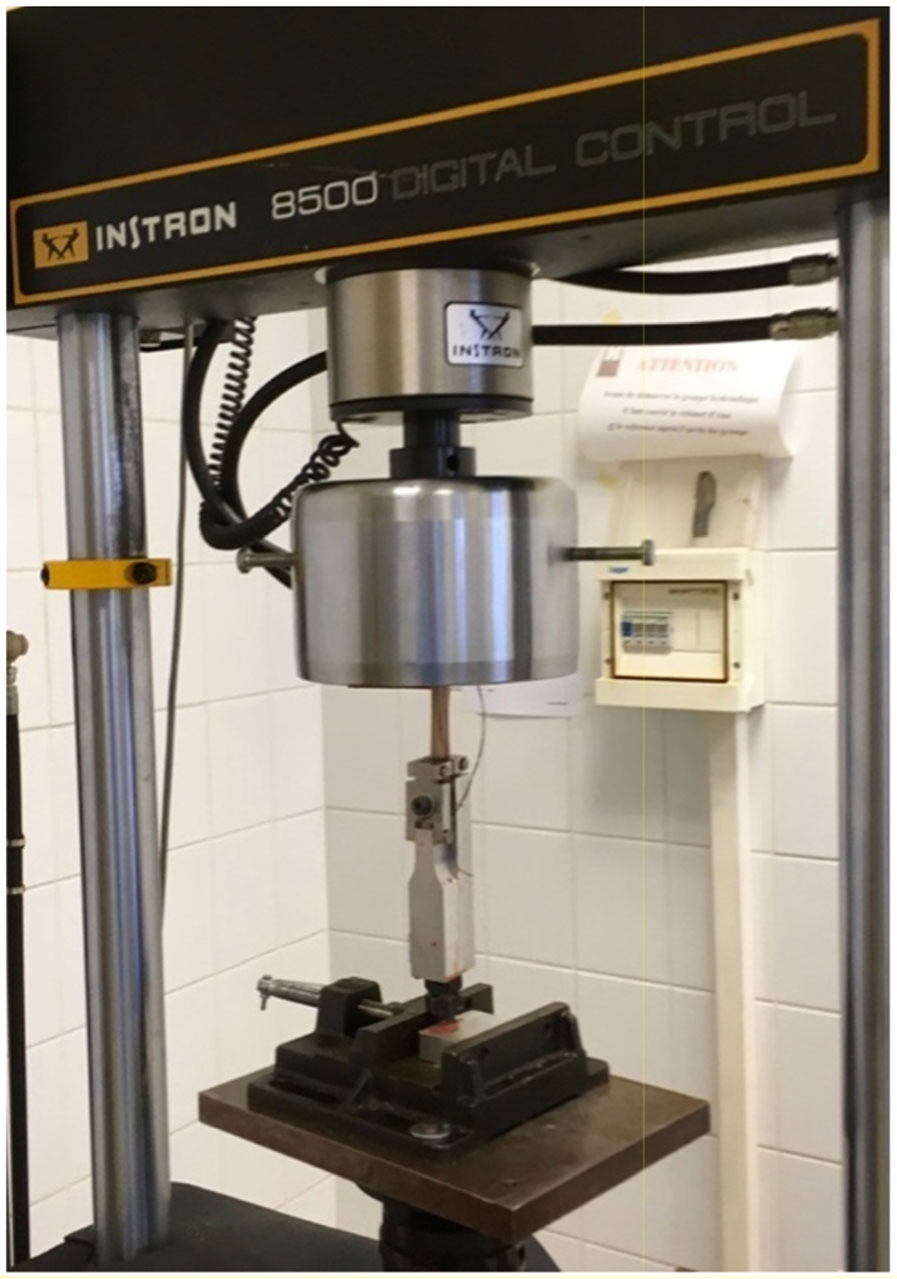
Loading a hamstring graft on to an Instron® device

The stiffness, ultimate load to failure and the mode of failure were recorded in testing to failure. The specimens were thawed for 12 h at room temperature (18 °C) before the mechanical testing. Each end of the tendon was then fixed in a grasping clamp over a length of 2.5 cm. Additional sandpaper was used to reinforce the grip of the clamp and avoid slippage during the test [11] (Fig. 4). The test was carried out on a servohydraulic materials test system (Instron® 8500 plus, Instron Corporation, High Wycombe, Buckinghamshire, UK). The specimens were installed vertically, so that the tendons were uniformly loaded in uniaxial tension.

The load to failure test was initiated by applying a 10-N preload followed by a tensile test at 10 mm/min constant speed until failure, using a standard and validated test protocol [8]. The load-elongation curve was recorded at 50 Hz, thus defining the stiffness (N/mm). The maximum load at failure was determined by the top of the curve. They were automatically measured by the software during the failure test.

The failure mode was defined by the level where the graft rupture occurred. Two types of rupture were distinguished. Failure of the graft in zone 1 meant a rupture at the grip interface of the graft on the clamp and failure in zone 2 was mid-substance failure between the two fixation points (Fig. 1).

### Statistical analysis

The data was collected using Excel 20A8 software (Microsoft Corp, Redmond, WA, USA) and XLSTAT 2018 (Addinsoft SARL, Paris, France).

Statistical analysis of the failure load and stiffness was performed using the Wilcoxon rank sum test with a 5% alpha risk. The level of significance was set at *p* < 0.05.

## Results

All the tests were performed without incident. No slippage of tendons in the grips during testing was detected. The values collected for each group are summarized in Table 1.

**Table 1:** Results of the biomechanical testing

	Knee	Length (mm)	Diameter (mm)	Maximal load to failure (N)	Stiffness (N/mm)	Failure mode
NSG	1	45	8	194.7	17.8	Zone 1
	2	90	9	186.7	17.9	Zone 1
	3	85	9	274.7	25.2	Zone 1
	4	76	10	244.7	29.0	Zone 1
	5	53	10	266.4	27.6	Zone 1
SG	1	48	8	139.0	13.2	Zone 1
	2	88	9	238.2	24.7	Zone 1
	3	83	10	194.8	22.5	Zone 1
	4	79	9	168.4	14.3	Zone 2
	5	56	10	235.7	24.0	Zone 1
		*p* value = 0.410	*p* value = 1.0			

*NSG* Non-sutured Group, *SG* Sutured Group

The working hypothesis was not confirmed; that is, adding stitches to the intra-articular portion of the four-strand hamstring-tendon graft does not increase its biomechanical properties. The maximal load to failure was 233.5 N ± 40.6 (186.7–274.5 N) for the NSG, 19.6% higher than for the SG which was 195.2 N ± 42.9 (139.0–238.2 N). The difference observed was not statistically significant (*p* = 0.188). Pull-out strengths for the non-sutured tendons were higher than for the respective paired, sutured tendons with one exception. The stiffness of the grafts for the NSG was 23.5 N/mm ± 5.3 (17.8–29 N/mm) and 19.7 N/mm ± 5.5 (13.2–24.7 N/mm) for the SG. Overall stiffness values for the NSG were 19% higher than those of the SG; however, the results were not statistically significant (*p* = 0.438, not significant) (Table 2).

**Table 2:** Statistical analysis

Group	Number	Maximal load to failure (N)		Stiffness (N/mm)	
		Mean	Standard deviation	Mean	Standard deviation
NSG	5	233.5	40.6	23.5	5.3
SG	5	195.2	42.9	19.7	5.5
		*p* value = 0.188		*p* value = 0.438	

*NSG* Non-sutured Group, *SG* Sutured Group

**Fig. 5:**
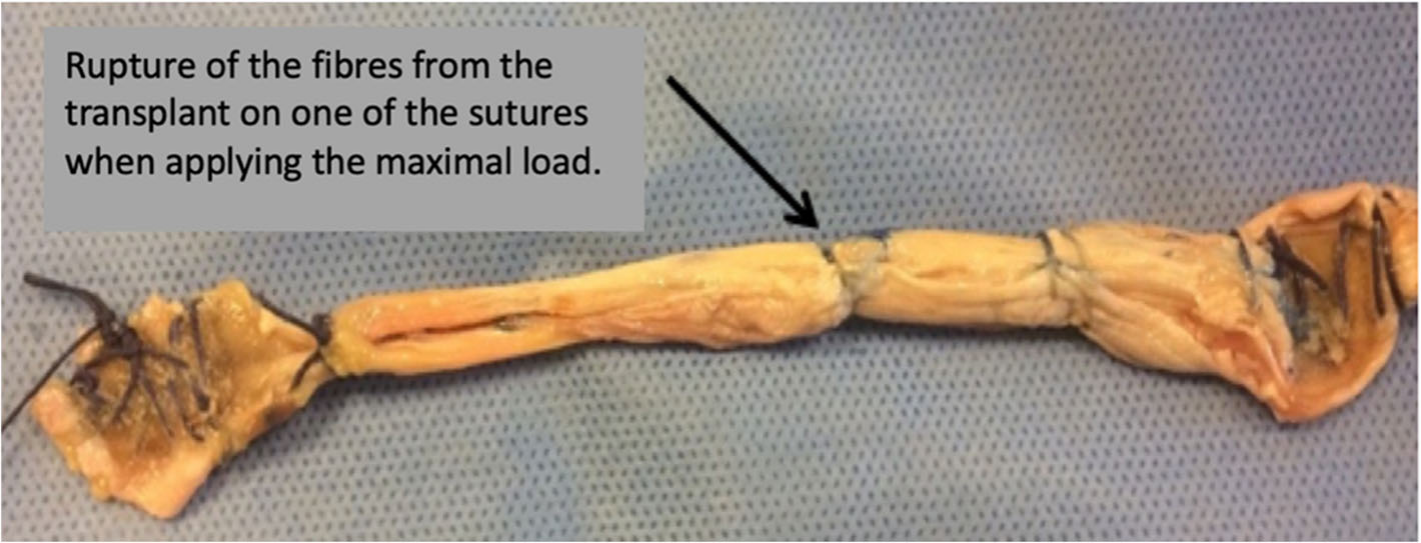
Rupture of the transplant of subject 4 from the Sutured Group (SG), in zone 2, on one of the stitches

Regarding the failure mode, the location of the rupture was closely analysed for all the grafts whilst performing the tests. In the SG group, the transplant rupture occurred in zone 1 in four cases and in zone 2 in one case. Concerning the latter, the rupture occurred by a dilaceration of the tendon fibres from one of the intra-articular stitches (Fig. 5). In the NSG, the rupture of the transplant occurred in zone 1 in all five cases. Failure in zone 1 was similar in both groups with a rupture that occurred at the grip interface of the clamp on the graft.

## Discussion

The working hypothesis was that additional stitches would increase the resistance of the transplants and, therefore, reduce their risk of breakage. The results of this study do not support that hypothesis. There was no significant difference between the two groups, even though the maximum loads to failure and stiffness were 20% greater in the NSG compared to those in the SG. Adding stitches to the intra-articular portion of the transplant does not seem to reinforce its properties. The biomechanical test outcomes even suggest that such added stitches could constitute points of weakness as observed in SG subject 4. This assumption is supported by all other transplant ruptures occurring at the fixation point and not on the graft mid-substance.

Considering all the different techniques, the iterative rupture rate of a reconstructed ACL is 6% according to the results of a meta-analysis by Jenny et al. [1]. Van Eck et al*.* have sought to determine the causes of these failures [11]. In 58% of cases, the rupture is due to functional insufficiency related to an elongation of the transplant. In 17% of cases, the rupture concerned the intra-articular portion. Whether the failure is an elongation or a rupture of the intra-articular portion, these figures underline the importance of the intra-articular portion of the graft, assuming of course that the positioning of the tunnels and the fixation technique are optimal.

The comparison with other study results was difficult because of the scarcity of similar publications. Furthermore, this study differs from previous ones because we investigated the use of isolated stitches instead of braided tendons or uniform sutures.

Nicklin et al. and Millet et al. studied the effects of continuous braiding of the four strands of a hamstring graft on the tensile strength and rigidity of the graft [7, 12]. They both concluded that the braiding of the transplant decreased significantly its strength and rigidity. Strength was shown to decrease by the square of the cosine of the twist angle.

Pailhé et al. found that a uniform suture of the patellar tendon over its entire length increased the graft strength [15]. They compared various ACL grafts; the quadrupled hamstring-tendon graft (QST4), the quadrupled semitendinosus graft (ST4), the quadrupled gracilis graft (G4) and patellar-tendon grafts (PT). All tendons were uniformly sutured over their entire lengths except for the right patellar tendons which were left in their native configuration. The ST4 construct had the highest mean load at failure followed in decreasing order by the GST4, G4, sutured PT and non-sutured PT. However, all their PT samples failed at the bone-tendon junction, whilst the GST4, ST4 and G4 failed at mid-substance. This discrepancy with our results can be explained by different experimental protocols but also by the different nature of transplants. The patellar transplant is a monobloc ligament whereas the hamstring graft is a four-time folded muscle tendon. Regarding the failure mode for the hamstring grafts, all their ruptures occurred in the sutured mid-substance. It is, thus, difficult to conclude that suturing reinforces the biomechanical properties of the grafts.

The results are also tempered by this study’s limitations. The maximum load values in the current study were much lower than those of published values and lower than the tension needed for walking or running. One step produces a tension of about 150 N on the ACL whilst walking and about 450 N whilst running. In order to meet the specifications of an ACL reconstruction, a transplant is expected to be able to support a load of 500 N. In this study, the tensile-strength breaking values are all well below 500 N. These values remain much lower than in other cadaveric study publications [10], with values up to 4546 N for maximum load and 490 N/mm for rigidity [12]. These differences can be explained by the advanced average age of the subjects, compared to patients featured in clinical practice [13]. It can also be explained by the fact that most of the biomechanical studies on tendons do not use the same measuring systems or protocols [7, 8, 10] and, therefore, with different loading rates, the viscoelastic properties of the tendons can be modified [10].

The experimentation on cadavers, and especially on elderly subjects, makes it difficult to extrapolate to clinical practice. However, all the data available in the literature is also experimental. Due to the cadaveric nature of this study, we could not take into account the biological phenomenon of transplant integration during the post-operative phase [14].

The samples were stored frozen and underwent two freeze-thaw steps. These steps do not significantly alter the samples’ biomechanical properties [4, 5]. This issue is still discussed by Clavert et al*.* who have shown that freezing has no influence on elongation during loading, but that the maximum breaking load and rigidity are reduced [8]. The explanation lies in the freeze-induced cellular dehydration of the tissues, which modifies their elastic properties. To overcome this phenomenon, the tendons were refrozen in compresses soaked in saline solution as recommended by current experimental protocols [10, 15].

We acknowledge some limitations to the technique used to fix the samples [3]. The challenge was to maintain a strong enough grip to prevent slipping when pulling, whilst avoiding the creation of an area of fragility at the attachment point [16, 17]. A clamp model [18] was used in this study and no failures related to tendon fixation were observed.

The sample used was small, a limitation inherent to the availability of cadaver parts. This restricted sample may explain the lack of significant difference between the two groups even though the results in the NSG were superior.

We believe, however, that this work has its own strengths as no study of this type has yet been published to our knowledge. Furthermore, the knees were paired and so were their own comparators. The weakness of this pairing is that we did not know the dominant character of the knees. Nevertheless, there were no significant differences in terms of length and thickness between the two groups.

## Conclusion

Whilst the initial hypothesis was not verified, nevertheless, the maximal load to failure and stiffness was approximately 20% higher when there were no intra-articular stitches compared to the situation where stitches were added to the intra-articular portion of the graft. This study remains important due to the absence of consensus on how to prepare the intra-articular portion of hamstring transplants whilst approximately 17% of iterative ACL ruptures are secondary to an intra-articular rupture of the transplant. This was a cadaveric pilot study and, therefore, whilst we are not able to extend our results to clinical practice, the outcomes would indicate the need for further development for this and related protocols deriving from the question of weakening the graft when adding stitches on its mid-substance. These results remain to be confirmed by further research.

## Data Availability

All data and materials of the study are available and will be kept for further reference.

## References

[1] Jenny JY, Besse J, Salle De Chou E, Diesinger Y. Devenir à Long Terme Des Ligamentoplasties Du Ligament Croisé Antérieur. In: L’Arthroscopie. UK: Elsevier Masson; 2015.

[2] Bonnin M, Amendola NA, Bellemans J, MacDonald SJ, Menetrey J, eds. The Knee Joint: Surgical Techniques and Strategies. Paris: Springer-Verlag; 2012.

[3] Hangody G, Pánics G, Szebényi G, Kiss R, Hangody L, Pap K. Pitfalls during biomechanical testing - Evaluation of different fixation methods for measuring tendons endurance properties. Physiol Intern. 2016;103(1):86–93. 10.1556/036.103.2016.1.8 27030630

[4] Woo SL, Orlando CA, Camp JF, Akeson WH. Effects of postmortem storage by freezing on ligament tensile behavior. J Biomech. 1986;19(5):399–404. 10.1016/0021-9290(86)90016-3 3733765

[5] Moon DK, Woo SL-Y, Takakura Y, Gabriel MT, Abramowitch SD. The effects of refreezing on the viscoelastic and tensile properties of ligaments. J Biomech. 2006;39(6):1153–57. 10.1016/j.jbiomech.2005.02.012 16549103

[6] Bahlau D, Clavert P, Favreau H, Ollivier M, Lustig S, Bonnomet F, et al. Mechanical advantage of preserving the hamstring tibial insertion for anterior cruciate ligament reconstruction - A cadaver study. Orthop Traumatol Surg Res. 2019;105(1):89–93. 10.1016/j.otsr.2018.11.014 30579723

[7] Cavaignac E, Pailhé R, Reina N, Murgier J, Laffosse JM, Chiron P, et al. Can the gracilis replace the anterior cruciate ligament in the knee? A biomechanical study. Int Orthop. 2016;40(8):1647–53. 10.1007/s00264-015-3027-9 26537395

[8] Clavert P, Kempf JF, Bonnomet F, Boutemy P, Marcelin L, Kahn JL. Effects of freezing/thawing on the biomechanical properties of human tendons. Surgical and radiologic anatomy: SRA . 2001;23(4):259–62. 10.1007/s00276-001-0259-8 11694971

[9] Höher J, Offerhaus C, Steenlage E, Weiler A, Scheffler S. Impact of tendon suturing on the interference fixation strength of quadrupled hamstring tendon grafts. Arch Orthop Trauma Surg. 2013;133(9):1309–14. 10.1007/s00402-013-1749-y 23836318

[10] Millett P, Miller B, Close M, I Sterett W, Walsh W, J Hawkins R. Effects of braiding on tensile properties of four-strand human hamstring tendon grafts. Am J Sports Med. 2003;31:714–17. 10.1177/03635465030310051301 12975191

[11] van Eck CF, Kropf EJ, Romanowski JR, Lesniak BP, Tranovich MJ, van Dijk CN, et al. Factors that influence the intra-articular rupture pattern of the ACL graft following single-bundle reconstruction. Knee Surg Sports Traumatol Arthrosc. 2011;19(8):1243–48. 10.1007/s00167-011-1427-y PMC3136704 21311861

[12] Handl M, Držík M, Cerulli G, Povýšil C, Chlpík J, Varga F, et al. Reconstruction of the anterior cruciate ligament: dynamic strain evaluation of the graft. Knee Surg Sports Traumatol Arthrosc. 2007;15(3):233–41. 10.1007/s00167-006-0175-x 16972110

[13] Noyes FR, Butler DL, Grood ES, Zernicke RF, Hefzy MS. Biomechanical analysis of human ligament grafts used in knee-ligament repairs and reconstructions. J Bone Joint Surg Am. 1984;66(3):344–52. 6699049

[14] Woo SL. Mechanical properties of tendons and ligaments. I. Quasi-static and nonlinear viscoelastic properties. Biorheology. 1982;19(3):385–96. 10.3233/bir-1982-19301 7104480

[15] Pailhé R, Cavaignac E, Murgier J, Laffosse J-M, Swider P. Biomechanical study of ACL reconstruction grafts. J Orthop Res. 2015;33(8):1188–96. 10.1002/jor.22889 25761203

[16] Shi D, Wang D, Wang C, Liu A. A novel, inexpensive and easy to use tendon clamp for in vitro biomechanical testing. Med Eng Phys. 2012;34(4):516–20. 10.1016/j.medengphy.2011.11.019 22189210

[17] Sharkey NA, Smith TS, Lundmark DC. Freeze clamping musculo-tendinous junctions for in vitro simulation of joint mechanics. J Biomech. 1995;28(5):631–35. 10.1016/0021-9290(94)00100-i 7775499

[18] Cheung JT-M, Zhang M. A serrated jaw clamp for tendon gripping. Med Eng Phys. 2006;28(4):379–82. 10.1016/j.medengphy.2005.07.010 16122965

